# The Development and Application of the Two Real-Time RT-PCR Assays to Detect the Pathogen of HFMD

**DOI:** 10.1371/journal.pone.0061451

**Published:** 2013-04-18

**Authors:** Aili Cui, Changping Xu, Xiaojuan Tan, Yan Zhang, Zhen Zhu, Naiying Mao, Yiyu Lu, Wenbo Xu

**Affiliations:** 1 National Institute for Viral Disease Control and Prevention, China CDC, Beijing, People’s Republic of China; 2 Zhejiang Provincial Centers for Disease Control and Prevention, Hangzhou City, Zhejiang Province, People’s Republic of China; University of North Carolina School of Medicine, United States of America

## Abstract

Large-scale Hand, Foot, and Mouth Disease (HFMD) outbreaks have frequently occurred in China since 2008, affecting more than one million children and causing several hundred children deaths every year. The pathogens of HFMD are mainly human enteroviruses (HEVs). Among them, human enterovirus 71 (HEV71) and coxsackievirus A16 (CVA16) are the most common pathogens of HFMD. However, other HEVs could also cause HFMD. To rapidly detect HEV71 and CVA16, and ensure detection of all HEVs causing HFMD, two real-time hybridization probe-based RT-PCR assays were developed in this study. One is a multiplex real-time RT-PCR assay, which was developed to detect and differentiate HEV71 specifically from CVA16 directly from clinical specimens within 1–2 h, and the other is a broad-spectrum real-time RT-PCR assay, which targeted almost all HEVs. The experiments confirmed that the two assays have high sensitivity and specificity, and the sensitivity was up to 0.1 TCID_50_/ml for detection of HEVs, HEV71, and CVA16, respectively. A total of 213 clinical specimens were simultaneously detected by three kinds of assays, including the two real-time RT-PCR assays, direct conventional RT-PCR assay, and virus isolation assay on human rhabdomyosarcoma cells (RD cells). The total positive rate of both HEV71 and CVA16 was 69.48% with real-time RT-PCR assay, 47.42% with RT-PCR assay, and 34.58% with virus isolation assay. One HFMD clinical specimen was positive for HEV, but negative for HEV71 or CVA16, which was identified as Echovirus 11 (Echo11) by virus isolation, RT-PCR, and sequencing for the VP1 gene. The two real-time RT-PCR assays had been applied in 31 provincial HFMD labs to detect the pathogens of HFMD, which has contributed to the rapid identification of the pathogens in the early stages of HFMD outbreaks, and helped to clarify the etiologic agents of HFMD in China.

## Introduction

Hand, Foot, and Mouth Disease (HFMD) is a common illness of infants and children under 10 years of age, and is characterized by fever, sores in mouth, and rash with blisters. Epidemiological data show that HFMD had an increasing trend in Southeast Asia in the past 20 years. It is well known that a big outbreak of HFMD occurred in Taiwan in 1998 [1]. In China, the number of HFMD cases and fatal HFMD cases has been increasing every year, and multiple major HFMD outbreaks caused by Human enterovirus 71 (HEV71) had occurred in several provinces in China. HFMD affects more than one million children and is responsible for several hundred children deaths every year, causing widespread concern in society.

HFMD is caused by human enteroviruses (HEVs), members of the Picornavirus family (single-stranded RNA, non-enveloped). HEV71 and coxsackievirus A16 (CVA16) are the two major causative agents of HFMD. It is worth noting that HEV71 may also cause severe neurologic diseases or significant fatalities, especially in young children [2], [3]. Hence, timely detection and differentiation of HEV71 specifically from CVA16 in the early phase of HFMD infection are important, which is helpful to reduce the deaths caused by HEV71. However, as the clinical symptoms of HEV71- and CVA16-associated HFMD are similar, the pathogen diagnosis largely depends on virus isolation and serotyping to differentiate HEV71 from CVA16 [4]. Using conventional RT-PCR strategy, specific detection of HEV71 or CVA16 has been reported [5], [6], [7], [8], [9]. However, the two-step RT-PCR process is time-consuming and easily prone to cross-contamination during the experiment, making it unsuitable for pathogen diagnosis in HFMD outbreaks. In recent years, real-time RT-PCR assay has gained wider acceptance for viral diagnosis in laboratories because of high sensitivity and specificity as well as rapid detection. Furthermore, it provides real-time monitoring of the amplification process through fluorescence emission [10].

To rapidly detect the pathogens in HFMD outbreak, a multiplex real-time hybridization probe-based RT-PCR assay was developed to detect and differentiate HEV71 specifically from CVA16 directly from clinical specimens within 1–2 h. In addition, as the etiologic agents of HFMD are very diverse, except for HEV71 and CVA16, other HEVs could also cause HFMD, such as CVA4–CVA7, CVA9, CVA10, CVB1–CVB3, CVB5, Echovirus 1 (Echo1), Echo4, and Echo19. To ensure detection of all etiologic agents of HFMD, another novel broad-spectrum real-time hybridization probe-based RT-PCR was also developed, which targeted almost all HEVs specifically. The two real-time RT-PCR assays were also evaluated in 213 HFMD clinical specimens, including stools, throat swabs, rectal swabs, and vesicles obtained from HFMD symptomatic individuals.

## Results

### Evaluations of the Sensitivity and Specificity of the Two Real-time RT-PCR Assays

The viral RNA of HEV71 and CVA16 derived from virus stocks of known titer was 10-fold serially diluted and used to determine the detection limits of the two real-time RT-PCR assays. The RNA of HEV71 and CVA16 samples with a concentration of 0.01–10^5^ TCID_50_/ml was extracted and tested in triplicates to determine the end-point dilution at which the positive amplification signal is obtained from all the three replicates. The results demonstrated that the sensitivity was 0.1 TCID_50_/ml for HEV71 and 0.1 TCID_50_/ml for CVA16 by multiplex real-time RT-PCR assay, and 0.1 TCID_50_/ml for HEVs in a broad-spectrum real-time RT-PCR assay.

All the available HEVs, including HEV71 and CVA16, and other RNA viruses commonly found in the respiratory tract, such as measles virus, rubella virus, mumps virus, influenza virus A1, influenza virus A3, and influenza virus B were used to determine the specificity of the two real-time RT-PCR assays. The results showed that the real-time RT-PCR assay for HEVs specifically detected all available enterovirus, but not any other RNA viruses tested. With regard to the multiplex real-time RT-PCR assay, the assay for HEV71 could specifically detect all the available HEV71 and none of CVA16, while the CVA16 assay also showed high specificity.

### Verifications of the Two Assays by Using Clinical Specimens

A total of 213 clinical specimens from HFMD clinical diagnosis patients during outbreaks were collected and tested, including 56 stools, 109 throat swabs, 38 rectal swabs, and 10 vesicles. All the samples were tested using the multiplex real-time RT-PCR assay for HEV71 and CVA16 and the broad-spectrum real-time RT-PCR assay for HEVs. The real-time RT-PCR results (Table 1) showed that the pathogens of these samples were mostly HEV71 (118) and CVA16 (30) among the 213 HFMD clinical specimens. With regard to the positive specimens for HEV71 or CVA16, the results of real-time RT-PCR for HEVs were also positive. On the other hand, only one HFMD clinical specimen was positive for HEVs, but negative for HEV71 or CVA16, which was identified as Echo11 by virus isolation, RT-PCR, and sequencing for the VP1 gene. The total positive rate of HEV71 and CVA16 was 69.48% (148/213) in the 213 HFMD clinical specimens.

**Table 1:** The real-time RT-PCR results of HEV71, CVA16, and HEVs for different types of HFMD clinical specimens.

Specimens types	No. of specimens	No. of HEV71-positive specimens	No. of CVA16-positive specimens	No. of HEVs-positive specimens
Stools	56	43	4	48*
Throat swabs	109	59	16	75
Rectal swabs	38	8	10	18
Vesicles	10	8	0	8
Total	213	118	30	149*

Note:

1. *Among 48 HEVs-positive specimens, one specimen was positive for HEVs, but negative for HEV71 or CVA16, which was identified as Echo11 by virus isolation, RT-PCR, and sequencing for the VP1 gene.

2. For the positive specimens for HEV71 or CVA16, the results of real-time RT-PCR for HEVs were also positive.

3. A total of 85 HEV71 and 16 CVA16 were identified using RT-PCR method among the 213 HFMD clinical specimens, which were lower than those of the real-time RT-PCR assay.

4. The multiplex real-time RT-PCR assay was shown to exhibit 100% specificity in detecting HEV71 and CVA16, which was confirmed by sequencing.

The 213 HFMD clinical specimens were also analyzed using direct conventional RT-PCR assay and virus isolation assay on human rhabdomyosarcoma cells (RD cells). A total of 85 HEV71 and 16 CVA16 were identified using RT-PCR method, while 65 HEV71 and 9 CVA16 were isolated using virus isolation assay. The total positive rates of RT-PCR and virus isolation for HEV71 and CVA16 were 47.42% (101/213) and 34.74% (74/213), respectively, when compared with that of real-time RT-PCR assay [69.48% (148/213)]. Thus, the sensitivity of real-time RT-PCR was noted to be significantly higher than both conventional RT-PCR and virus isolation.

From the results of the multiplex real-time RT-PCR assay and conventional RT-PCR assay for HEV71 and CVA16, it can be observed that no amplification occurred for the other enterovirus serotypes. The amplicons generated by real-time RT-PCR for HEV71 and CVA16 were verified by DNA sequence analysis and blast results. Thus, it can be concluded that the multiplex real-time RT-PCR assay has 100% specificity in detecting HEV71 and CVA16.

### Applications of the Assays in HFMD Laboratory Network

The one-step multiplex real-time RT-PCR assay for HEV71 and CVA16 and the broad-spectrum real-time RT-PCR assay for HEVs had been applied in 31 provincial labs to detect the pathogens of HFMD. Based on the laboratory surveillance data obtained from six provincial HFMD labs in China, up to Dec 13, 2009, a total of 3834 HFMD cases detected by the two real-time RT-PCR methods were reported: 2046 cases were identified as HEV71(53.36%), 1030 cases were CVA16 (26.86%), and 758 cases were identified as other enterovirus(19.77%), respectively. The 2012 laboratory data obtained using the two real-time RT-PCR assays showed that HEV71 and CVA16 were the main pathogens in HMFD cases; however, CVA2, CVA4, CVA8, CVA9, CVA10, CVB3, Echo3, Echo9, Echo19, Echo25, and Echo30 were also detected in several hundreds of HFMD clinical specimens, which were confirmed by sequencing.

## Discussion

The classical “gold standard” diagnosis for HEV71 and CVA16 is by cell culture, followed by neutralization tests with serotype-specific antisera [4]. However, this requires 2–3 weeks of growth and neutralization of the viral isolates. Furthermore, antigenic typing could be hindered by nonneutralizable viruses because of aggregation, antigenic drifts, or the presence of multiple viruses in the specimens [11]. The development of PCR techniques has contributed significantly to laboratory diagnosis of viral infections in terms of sensitivity, specificity, and the rate of detection in comparison with the cell culture method.

The real-time PCR assays developed in recent years have many advantages, such as improved sensitivity, specificity, and reproducibility, and have been applied to the detection of a range of different respiratory viruses [12], [13]. An important aspect of this method is that the reaction and detection take place in a closed tube, which can effectively avoid contamination, and no post-PCR handling, such as agarose gel electrophoresis, is required.

TaqMan probe technology has been used successfully in the detection of a number of viruses. In this study, the multiplex real-time RT-PCR assay for HEV71 and CVA16 and the broad-spectrum real-time RT-PCR assay for HEVs were developed for rapid detection of the pathogens causing HFMD. The primers and probes were designed, and the reaction parameters and running conditions of real-time RT-PCR assays were optimized and evaluated to ensure accurate detection of HFMD in 213 clinical specimens. The VP1 region of the HEV71 and CVA16 was selected because it possessed high degree of antigenic and genetic diversity to distinguish the HEVs serotypes [6], [14]. For HEVs, the 5′-UTR was selected for detection of all the HEVs serotypes.

In the multiplex real-time RT-PCR assay for HEV71 and CVA16 and the broad-spectrum real-time RT-PCR assay for HEVs, FAM and HEX (VIC) were used in different probes. As these were widely used reporters for TaqMan probe chemistry, the two real-time TaqMan RT-PCR assays are versatile and could be applied across other real-time PCR systems.

The two real-time RT-PCR assays showed high sensitivity for HEV71, CVA16, and HEVs in this study, which were able to detect as low as 0.1 TCID_50_/ml of HEV71, 0.1 TCID_50_/ml of CVA16, and 0.1 TCID_50_/ml of HEVs. The entire procedure for real-time RT-PCR detection required only 2 h, which was more rapid than the tissue culture method and conventional RT-PCR assay. In addition, as the experimental run protocol of the multiplex real-time RT-PCR assay for HEV71 and CVA16 is similar to that of the broad-spectrum real-time RT-PCR assay for HEVs, the two real-time RT-PCR assays could be simultaneously run for detection in the ABI7500 instrument. Combined with the use of these two assays, the pathogens of HFMD could be quickly determined in the early stage of HFMD outbreaks.

HFMD laboratory network, including 31 provincial labs and 331 prefectural labs, has been established for the pathogens surveillance of HFMD due to the severe situations of HFMD in China in the recent years. The one-step multiplex real-time RT-PCR assay for HEV71 and CVA16 and broad-spectrum real-time RT-PCR assay for HEVs have been applied in 31 provincial labs to detect the pathogens of HFMD. The widespread application of the two real-time RT-PCR methods in the HFMD laboratory network will contribute to the rapid identification of HEV pathogens in the early stages of HFMD outbreaks, and will help clarify the etiologic agents for HFMD in China.

## Materials and Methods

### Virus Strains and Clinical Samples

The virus strains used for determining the sensitivity and specificity of the newly developed assays were as follows: Coxsackieviruses A group (CVA4, CVA16, CVA21, and CVA24), Coxsackieviruses B group (CVB1, CVB3, Echo3, Echo6, Echo7, Echo14, Echo20, Echo30, and HEV71), and Poliovirus type 1 (PV1), PV2, and PV3 (Sabin strains). For HEV71, the virus strains of genotype A and subgenotype B3 and subgenotype C4 were collected for detection. All of them were identified by neutralization test or VP1 gene sequencing. In addition, influenza A1, A3, and B, measles virus, rubella virus, and mumps virus were also selected for this study.

In addition, 213 HFMD clinical specimens, including 56 stools, 109 throat swabs, 38 rectal swabs, and 10 vesicles were collected from 179 HFMD patients by clinical diagnosis during HFMD outbreaks. Among them, 34 paired specimens were collected from 34 HFMD patients, including 22 pairs of throat swabs and rectal swabs, 8 pairs of throat swabs and vesicles, and 2 pairs of throat swabs and stools. These specimens were simultaneously tested by the two real-time RT-PCR assays, RT-PCR assay, virus isolation, and the VP1 gene sequencing assay. All the specimens and virus strains were stored at −80°C until further use.

### Ethics Statement

This study did not involve human participants or human experimentation; the only human materials used were collected for public health purposes from patients with clinically suspected HFMD. All written informed consents for the use of the clinical samples were obtained from the patients or guardians on behalf of the minors/children participants involved in this study. This study was approved by the second session of the Ethics Review Committee of the Chinese CDC.

### Primers and Probes Designed for HEV71 and CVA16 in a Multiplex Real-time Hybridization Probe-based RT-PCR Assay

As the VP1 gene of HEVs appears to correlate with serotype and is used to distinguish the HEVs serotypes, this region was targeted to develop the multiplex real-time RT-PCR assay for simultaneously detection of HEV71 and CVA16. The VP1 nucleotide sequences of HEV71 and CVA16 strains from GenBank were analyzed. Using BLAST (http://www.ncbi.nlm.nih.gov/BLAST) and DNASTAR program, highly conserved VP1 sequences of HEV71 or CVA16 were utilized to design primers and probes. A pair of primers (HEV71YGF and HEV71YGR) and a hybridization probe [HEV71YG(HEX)PB] were designed for specific detection of HEV71, and another pair of primers (CVA16YGF and CVA16YGR) and a hybridization probe (CVA16YGPB) were designed for specific detection of CVA16. The TaqMan probes of HEV71 and CVA16 were labeled with HEX and FAM fluorescence dyes at their 5′-ends, respectively, and with BHQ1 at the 3′-end. In addition, it must be emphasized that there was no detection channel for HEX fluorescence in the ABI7500 instrument. As VIC and HEX were very similar in the fluorescence emitted, VIC could be used for detection, instead of HEX in the ABI7500 instrument. The sequences and details of primers, probes, and amplicons of HEV71 and CVA16 are listed in Table 2.

**Table 2:** The sequences of the primers and probe for HEV, HEV71, and CVA16.

Type	Name	Nucleotide sequence (5′–3′)	Position in Genome
Primer	EV(YG)F:	GGCTGCGYTGGCGGCC	361–376 a
Primer	EV(YG)R:	CCAAAGTAGTCGGTTCCGC	536–554 a
Probe	EVTY(YG)PB:	FAM -CTCCGGCCCCTGAATGCGG -BHQ1	450–468 a
Primer	HEV71YGF	TGATTGAGACACGSTGTGTYCTTA	2626–2649 a
Primer	HEV71YGR	CCCGCTCTGCTGAAGAAACT	2682–2701 a
Probe	HEV71YG(HEX)PB	HEX - TCG CAC AGC ACA GCT GAG ACC ACT C - BHQ1	2652–2676 a
Primer	CVA16YGF	GGGAATTTCTTTAGCCGTGC	2686–2705 b
Primer	CVA16YGR	CCCATCAARTCAATGTCCC	2771–2789 b
Probe	CVA16YGPB	FAM -ACA ATG CCC ACC ACG GGT ACA CA - BHQ1	2725–2747 b

Note:

1. “a” indicates the sequence position of the primers and probes referring to strain BrCr of HEV71 (GenBank accession number U22521); “b” indicates the sequence position of the primers and probe referring to strain G-10 of CVA16 (GenBank accession number U05876).

2. The lengths of real-time RT-PCR products for HEV71, CVA16, and HEV were 76, 104, and 194 nt, respectively.

3. The primers and probes of HEV71, CVA16, and HEV have been applied for national invention patent in China, and the numbers of the patents are ZL200810063097.5, ZL200810121454.9, and ZL200810121453.4, respectively.

### Primers and Probe Designed for HEVs in a Broad-spectrum Real-time Hybridization Probe-based RT-PCR Assay

It is well known that the etiologic agents related to HFMD are very diverse. To ensure detection of all etiologic agents of HFMD, a broad-spectrum real-time hybridization probe-based RT-PCR was developed, which targeted almost all HEVs specifically. Alignment studies of the sequenced Echo7, Echo12, Echo14, Echo15, Echo21, Echo28, Echo32, and Echo34 revealed highly conserved sequences within the 5′-noncoding region (5′-NCR). To detect almost all HEVs that caused HFMD, the primers [EV(YG)F and EV(YG)R] and probe [EVTY(YG)PB] in this conserved region were selected to obtain a broad specificity for the genus *Enterovirus*. Using these primers and probes, a broad-spectrum real-time RT-PCR assay was developed, which could detect nearly the entire HEVs group. The sequences and details of the primers, probes, and amplicons for HEVs are listed in Table 2.

### Real-time RT-PCR Reaction Parameters and Running Conditions

Both the multiplex real-time RT-PCR assay for EV71 and CVA16 and the broad-spectrum real-time RT-PCR assay for HEVs were developed into one-step assays, in which reverse transcription was a part of the process and extracted RNA was used as template. Reagents from one-step Prime Script RT-PCR Kit (TAKARA, Japan) were used for preparing master mixtures according to the guidelines of the manufacturer.

To facilitate the operations, the real-time RT-PCR reaction parameters and running conditions were optimized to be very similar for the two real-time RT-PCR assays. The RT-PCR mastermixture of 25-µl volume consisted of 12.5 µl of 2× one-step RT-PCR buffer, 0.5 µl of Ex Taq HS (5 U/µl), 0.5 µl of RT-Enzyme Mix II (5 U/µl), 0.6 µl of each primer (20 pM for each primer), and 0.3 µl of each probe (20 pM for each probe), and 5 µl of RNA template and RNase-free water to give a total volume of 25 µl for each reaction tube. The final concentration of the primers and probes were 12 and 6 pmol/25 µl of reaction, respectively. An optimized experimental run protocol for the ABI7500 instrument was used as follows: reverse transcript program (42°C for 30 min), denaturation program (95°C for 2 min), amplification program repeated 40 times (95°C for 5 s, 55°C for 35 s), and fluorescence measurement at 55°C of each cycle. It is worthwhile to note that the primers of HEV71YGF, HEV71YGR, CVA16YGF, and CVA16YGR, and the probes of HEV71YG (HEX)PB and CVA16YGPB were simultaneously used in the real-time RT-PCR assay for HEV71 and CVA16, and the primers of EV(YG)F and EV(YG)R and the probe of EVTY(YG)PB were used for the detection of HEV. Moreover, FAM and VIC/HEX fluorescence were simultaneously measured at 55°C of each cycle in HEV71 and CVA16 assay, and only FAM fluorescence was measured for HEVs.

### Virus Isolation

The 213 clinical specimens were separately inoculated into RD cells. The virus isolates were harvested when the cytopathic effect (CPE) affected 75∼100% of the monolayer. If no CPE was observed in the cultures, the virus isolates were further cultured up to three passages. The positive virus isolates were identified by using RT-PCR methods and sequencing. The virus isolates were frozen and thawed twice and then stored at −80°C.

### RNA Extraction

All the clinical specimens, virus strains, and positive virus isolates were extracted in a set of standard PCR laboratories. The throat swabs, rectal swabs, and vesicles stored in viral transport media (VTM) were processed for RNA extraction directly. A 10% stool suspension was made by adding 0.5 g of stool to 5 ml of 1% PBS. The suspension was then centrifuged at 12,000×*g* for 10 min, and the upper suspension was used for RNA extraction. QIAamp Viral RNA Mini Kit (Qiagen, Hilden, Germany) was used for RNA extraction in the development of the assay and RNA extraction from clinical specimens according to the manufacturer’s instructions. The RNAs were eluted with 40 µl of diethyl pyrocarbonate-treated water and kept at −80°C until further use for real-time RT-PCR.

### RT-PCR Assays and Sequence Determination

The 213 clinical specimens and positive virus isolates were subjected to RT-PCR assays, and the partial VP1 gene of HEV71 and CVA16 was amplified as previously described [15].The amplification products were purified using a QIAquick Gel Extraction Kit (Qiagen), and the amplicons were bi-directionally sequenced using an ABI PRISM 3100 Genetic Analyzer (Applied Biosystems, Hitachi, Japan).
